# Occurrence of Microplastics in Commercial Seafood under the Perspective of the Human Food Chain. A Review

**DOI:** 10.1021/acs.jafc.0c01209

**Published:** 2020-04-20

**Authors:** Raffaelina Mercogliano, Carlo Giacomo Avio, Francesco Regoli, Aniello Anastasio, Giampaolo Colavita, Serena Santonicola

**Affiliations:** † Dipartimento di Medicina Veterinaria e Produzioni Animali (MVPA), Università Federico II di Napoli, Via F. Delpino, 1, 80137 Napoli, Italy; ‡ Dipartimento di Scienze della Vita e dell’Ambiente (DiSVA), Università Politecnica delle Marche, Via Brecce Bianche, 60131 Ancona, Italy; § Dipartimento di Medicina e Scienze della Salute V.Tiberio, Università del Molise, Via Francesco De Sanctis, 1, 86100 Campobasso, Italy

**Keywords:** microplastic, marine food web, commercial seafood, human health

## Abstract

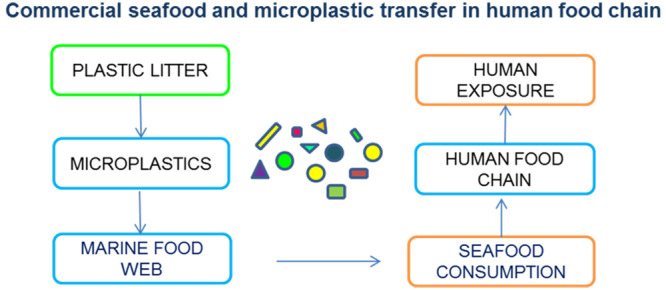

The occurrence of microplastics in the marine ecosystem and aquatic organisms, their trophic transfer along the food web, and the identification of seafood species as suitable indicators have become a research priority. Despite the high quantity of research in this field, a comparison between the available data and an appropriate risk assessment remains difficult. In this perspective, as an innovative approach, the association of the feeding strategies of commercial seafood and the microplastic level was considered. Further research to assess the occurrence of microplastics in the marine food web, the long-term effects on animals and humans, and the health implications is needed.

## Introduction

During the past few years, the environmental ubiquity of microplastics (MPs), differently shaped particles with a grain size of 0.1–5000 μm, has become a critical concern. MPs may be found as fibers, fragments, spheroids, beads, granules, pellets, or flakes, which may result directly from human activity (primary MPs) or the fragmentation of larger plastic objects (secondary MPs) by mechanical, biodegradation, and photodegradation.^1−3^

**Figure 1:**
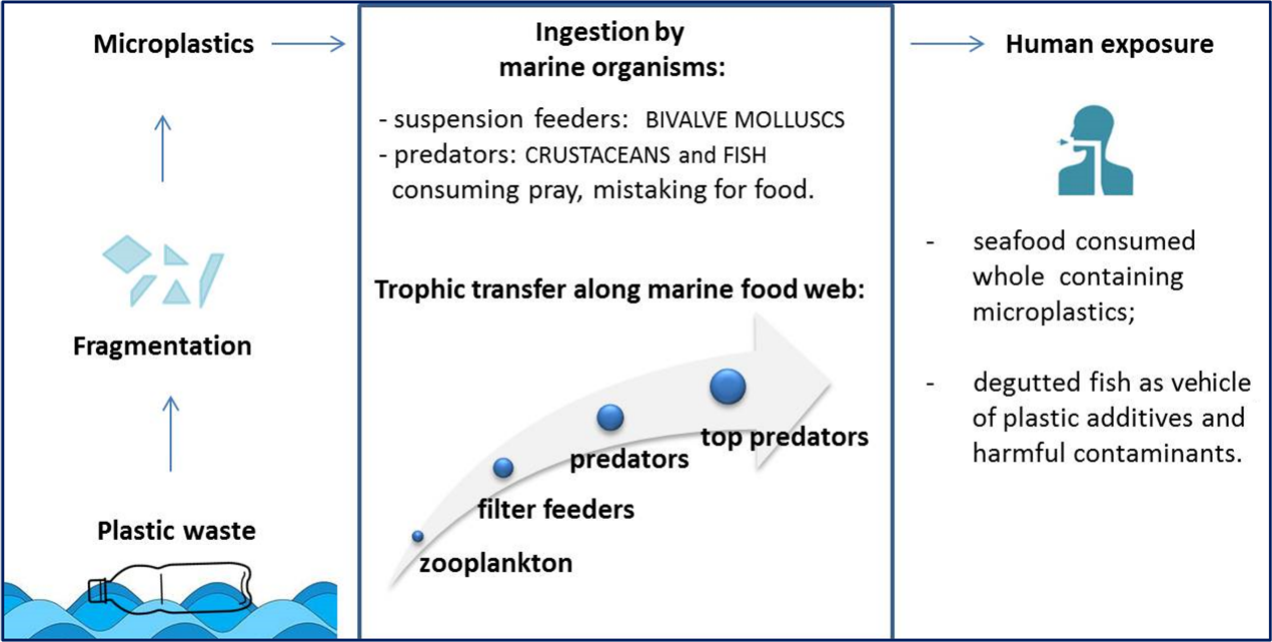
Microplastic transfer along marine trophic levels and human exposure.

MPs may impact human health, particularly through the contamination of the food chain. They can be ingested by marine organisms and transferred from one trophic level to the next (Figure 1).^2,4,5^ Fish and seafood represent one of the most important routes of exposure for humans through the diet, associated also to nonmarine sources, such as honey, salt, sugar, and beer.^5^ MPs may also leach plastic additives or adsorb contaminants from the marine ambient. These chemicals, including persistent organic pollutants (POPs), polycyclic aromatic hydrocarbons (PAHs), and heavy metals, may be transferred and accumulated by marine organisms, undergoing biomagnification along the food chain.^6^

In light of the above, different methods to determine and identify MPs have been developed. The assessment of the occurrence of MPs in marine biota and their trophic transfer is first based on particle isolation and identification.^6^ Methods for extracting and characterizing MPs from organic tissues include several steps.^7,8^ Among the different digestion methods of biological material, the acidic, basic, or oxidizing treatments may degrade plastic polymers which are pH-sensitive. The application of enzymatic digestion seems to be a reliable method but with the disadvantage of high costs.^1,9^ Recently, a protocol has been applied combining a density gradient separation and the addition of hydrogen peroxide (15%), to allow the digestion of biological material facilitating the plastic detection.^1^ The majority of MPs, identified through spectrometric characterization (μFT-IR and μRAMAN analysis), in seafood are composed by polypropylene (from the fragmentation of soft plastic bags and food packaging), polyvinyl chloride (plastic coatings for the freight transport and bottle tops), polyethylene (fishing gear), and polyethylene terephthalate (water bottles).^2^ Despite the high quantity of research in this field, the comparison between the available data remains difficult due to the use of a wide range of methodologies and reporting units.^7,8,10^

Considering the MP ubiquity, numerous attempts have been made to assess the effects not only on the environment but specifically on biota and humans. The exposure to plastic debris may cause in marine organisms physical and mechanical damage, inflammation, obstruction of the gastrointestinal tract (GI), and impairment of immune and stress response, growth rate, and damage repair.^4,11^ Plastic pollution may represent a potential threat for the oceans, living organisms, and food webs.^10^ Nevertheless, studies on the effects of MPs on human health under the perspective of the food chain are scarce. The study provides a new perspective about the occurrence of MPs in commercial seafood describing the MP trophic transfer along the marine food web, associated with fish feeding strategies, and the implications for food safety and consumer health.

## Occurrence and Trophic Transfer of Microplastics into the Marine Food Web

The contamination of the sea environment is particularly relevant near the population centers, related to land-based sources (wastewater, industrial plants, and other human activities) and marine sources (navigation, fishing, and oil platforms).^12^

The density characteristics of each polymer allow MPs to disperse differently in the water column and sediments. Also, the species, the season of sampling, and the sex of animals could affect the levels of ingested MPs at different trophic levels of the marine web.^13^ MPs can accumulate in the sea sediment surface, in phytoplankton at the base of the marine food web, and in some subsurface layers and can be ingested by species (e.g., crustaceans) living in the benthic zone.^2^ At the next level, MPs may be detected in zooplanktonic species (*Chaetognatha*, *Copepoda*, *Salpida*), which sustain a large group of carnivorous marine organisms (sardines, herring, menhaden, octopuses) and many fish feeding on small invertebrates. Zooplanktonic herbivorous species, such as jellyfish, larval stages of fish, barnacles, and mollusks, feast on the sea plants and the ocean surface waters, introducing small plastics with an exposure level depending on species, stage of life, and particle dimensions. Finally, at higher trophic levels, invertebrates (*Polychaeta*, *Crustacea*, *Echinodermata*, *Bryozoa*, *Bivalvia*) and vertebrates (benthic and pelagic fish, marine mammals, and seabirds) may ingest MPs directly or indirectly while consuming prey.^9,13,14^In particular, fish may ingest plastic material through different feeding strategies that could lead to an increase or decrease in the ingestion of MPs. Generally, herbivores are opportunistic and flexible in their feeding habits, while piscivores may ingest other fish, as main prey items, or show a filter-feeding behavior. This feeding strategy is used by piscivores and planktivorous fishes on small prey and may expose them to high MP ingestion levels. In predators such as *Thunnus thynnus*, *Thunnus alalunga*, and *Xiphias gladius*, it is possible to distinguish primary and secondary exposure pathways, respectively, by MP ingestion during hunting and through the prey.^13,15^ MP biomagnification through the trophic transfer into the marine web has been investigated in various commercial species such as in crabs (*Carcinus maenas*) through mussel ingestion, in shrimps from zooplankton, and in fur seals consuming pelagic fish. When commercial seafood is considered, the bioaccumulation and biomagnification of MPs in the marine web might pose a threat under the perspective of the human food chain representing a serious issue to food safety.^4,16^

## Influence of the Feeding Strategies of Seafood on the Occurrence of Ingested Microplastics

**Figure 2:**
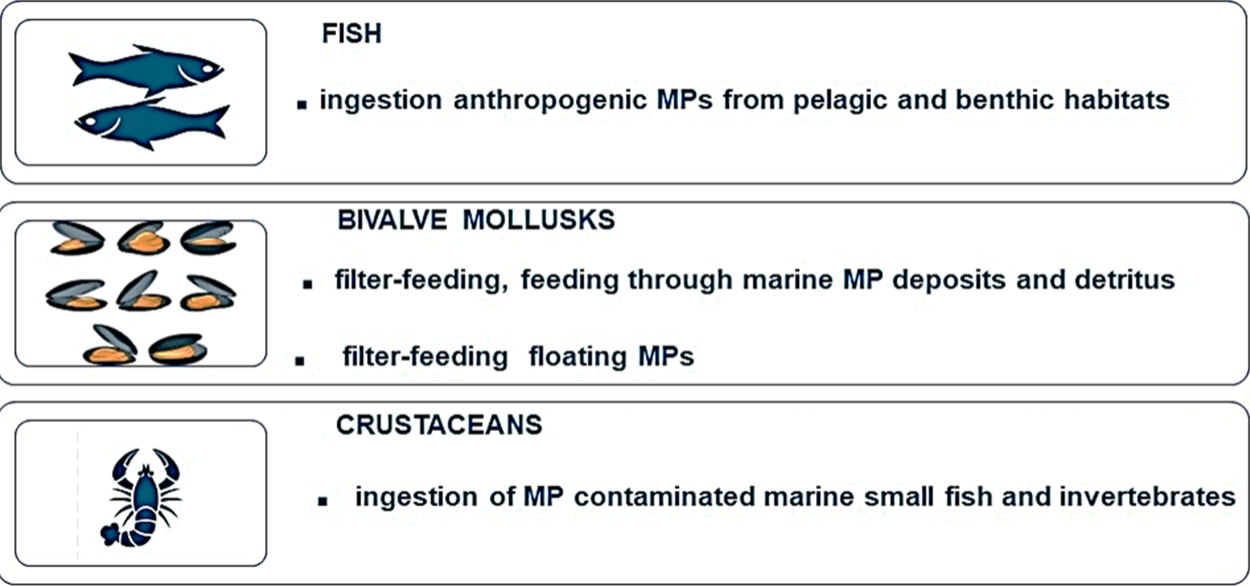
Relationship between fish, bivalve, and crustacean feeding strategies and the occurrence of microplastics.

Feeding strategies of seafood may influence the levels and the type of ingested MPs and their distribution along the trophic levels of the marine food chain (Figure 2).^17^

- *Commercial fish species* – The occurrence of MPs in the guts and/or the tissues of fish of commercial interest has been documented (Table 1).^2,5^ According to the feeding strategies, some fish have a highly selective diet and only rarely may eat plastics. However, reducing the MP size, fish ingestion may increase due to their inability to distinguish between food and nonfood particles.^17^ On the contrary, opportunistic feeder fish such as the Atlantic cod may hunt and feed on a wide variety of prey. This aspect makes them more exposed to the ingestion of anthropogenic particles dispersed in the water column.^18^ MPs have been detected in migrating commercial fish (*Thunnus thynn*us), shelf-sea species with seasonal migrations (*Dicentrarchus labrax*), or, also, stationary coastal fish (*Pleuronectes platessa*). Seasonal influence on the feeding strategies has been observed in *Scomberomorus cavalla* and *Rhizoprionodon lalandii* from Brazil, which showed a greater MP intake in October than March.^5^ MPs are also detected in Mediterranean fish of great commercial importance,^1^ such as *Engraulis encrasicolus* and *Sardina pilchardus*, which are often consumed whole. Differences in the feeding behavior among the two species are responsible for the different MP content. Anchovies are selective feeders, while sardines, as filter feeders, are unable to select the ingested particles.^10,11^ During the spring and summer months (the spawning period), the females may ingest and filter indiscriminately small planktonic organisms and floating MPs (mistaken as prey) migrating toward surface water.^10^
- *Bivalve mollusks* – Marine invertebrates may ingest MPs according to different feeding strategies, like filter and deposit feeders and detritivores.^16^ Commercial bivalve mollusks can filter and retain MPs of different sizes, at levels depending on plastic particle concentration and distribution in the seawater.^12^ MPs (2–10 μm) are then transferred from the gut in the circulatory system for longer-term storage. A number of 3–5 fibers/10 g of bivalves has been observed in different species of mussels (*M. edulis and galloprovincialis*) from Belgium.^5^ In commercial bivalves from China, MPs vary from 2 to 11 items/g (5–5000 μm) and from 4 to 57 items/bivalve.^4,5^ Although there are no significant differences in MP content between wild and cultured mussels, the latter may be exposed also through the use of plastic ropes and nets.^2,19^ The application of a depuration treatment allows the excretion of all or a part of the biggest ingested MPs in mussels and oysters, respectively.^19^ However, in scallops (*Placopecten megallaniccus*, *Crassostrea virginica* o Gmelin), only larger, longer, and denser particles are retained, not allowing MP excretion.^20^
- *Crustaceans* – Also, commercial crustaceans exhibit a wide range of feeding techniques. Their uptake of MPs may be both accidental and also related to the active collection during the feeding.^19^ Swimming crustaceans may ingest more particles than those living on the seabed. Copepods and tiny shrimps, as filter feeders, may be exposed to MPs through plankton and suspended materials. In other cases, crustaceans may be opportunistic feeders (*Crangon crangon*) or active hunters of small fish and other organisms (crabs and lobsters).^20^ Norway lobsters may ingest MPs when they are fed with pieces of fish seeded with plastic strands, even if this does not reflect the natural trophic level.^12,19^ MPs are determined in lobsters at different levels related to their sex. Female lobsters retain more MPs than males, probably due to less frequent molting. The prey consumed by shrimps (mollusks, arthropods, young fish) may contain MPs which are accumulated in the digestive tract. Considering that shrimps are consumed whole, without removing the GI tract, more attention should be paid to their contribution to human exposure.^20^

**Table 1:** Occurrence of MPs in Commercial Marine Seafood Included in the FAO List-2016 (Barboza et al., 2018^5^)

species name	MP levels^a^	size range (μm)	sample type^b^	MP types	cathfish location
*Clupea harengus*	566 (2%)	>1000	GI	fibers, fragments	North Sea
*Decapteru smacrosoma*	17 (29%)	>500	GI	fragments, styrofoam	Indonesia
					Eastern from local market
*Decapterus muroadsi*	20 (80%)	5000	gut	fragments	South Pacific
*Engraulis japonicus*	64 (77%)	10–500	GI	fragments, bead, filament, foam	Tokyo Bay
*Gadusmorhua*	80 (13%)	>1000	GI	fibers, fragments	North Sea
	74 (1.4%)	<5000	GI	fibers, fragments, film	Baltic Sea
	205 (2.4%)	2800–4200	GI	fragments	Coast of Canada
	302 (18.8%)	5000–20,000	stomach	fibers, fragments, granule, film	Norwegian Coast
*Micromesistius poutassou*	27 (51.9%)	1000–2000	GI	fibers, fragments, beads	English Channel
*Sardinella longiceps*	10 (60%)	500–3000	gut	fragments	Indian Coast
*Sardina pilchardus*	99 (19%)	10–5000	GI	fragments, line, film, pellet	Adriatic Sea
*Scomberomorus cavalla*	8 (62.5%)	1000–5000	stomach	pellets	Northeastern Brazil
*Scomberj japonicas*	7 (71%)	>9.07	GI	fibers, hard plastic, nylon	Mediterranean Sea
	35 (31%)	217–4810	GI	fragments, fibers	Portuguese Coast
	30 (3.3%)	≤2100	gut	fragment	Southeast Pacific Ocean
*Scomber scombrus*	13 (31%)	217–4810	GI	fragments, fibers	Portuguese Coast
*Sprattus sprattus*	515 (18.8%)	100 – >5000	GI	fibers, fragments	Baltic Sea

a Number of analyzed samples and % value of samples containing MPs.

b GI: gastrointestinal tract.

## Seafood as Bioindicators of Microplastic Pollution and Food Safety

MP abundance in the marine habitat and biota calls for identifying adequate indicator species to assess the MP pollution, biotic impact exposure, ecological, and human risks.^1,4,17^ Mussels (*Mytilus* spp.) are considered one of the main indicator species, since they are widely distributed in several marine areas and may tolerate different environmental conditions.^4,8^ In addition, as filter feeders, they are directly exposed to MP contamination, showing a positive correlation between particle occurrence in their tissues and the surrounding water. Finally, mollusks are consumed whole and represent the most important source of MP human exposure through the diet. Thus, they are suitable indicators for MP contamination and human food safety.^4,8^ For the same reasons, the widespread invasive species Asian clam (*Corbicula fluminea*) has been proposed as a bioindicator in freshwater systems.^21^

Because of their strict connection with the seabed, demersal fish are considered small-scale indicators of the benthic habitat contamination. Generally, it is advantageous to use these species, since marine sediments have been identified as an important sink and ultimate end point for MPs.^8^ High levels of MP have been detected in fish such as *Mullus barbatus* and *Solea* spp., which live on muddy and sandy bottoms. *Merluccius merluccius*, an important commercial species, is also considered a suitable indicator because it represents the trophic link between pelagic and demersal environment.^11,17,22^ Although demersal fish are usually eviscerated before the consumption, MPs in the fish stomachs might be transferred to edible tissue representing a risk for human health.^11^ On the other hand, also sardines (*S. pilchardus*) and anchovies (*E. encrasicolus*), usually consumed whole, have been proposed as small-scale species indicators both of MP contamination in open waters and human exposure. Sardines and anchovies are important commercial seafood also composing the main diet for pelagic predators in the Mediterranean Sea.^10,23^

*Thunnus alalunga* and *Coryphaena hippurus*, as pelagic predators, are considered bioindicators at a medium scale for monitoring the MP contamination along the trophic web. Also, the occurrence of chemicals in fish tissues has been considered as an indicator of MP human exposure. Therefore, large pelagic predators should be considered as key species, since they may also be subject to chemical bioaccumulation.^11^

The studies on the spatiotemporal correlations between the occurrence of MPs in a marine habitat and in living organisms are still at a preliminary stage.^8^ However, the identification of species as suitable indicators has become a research priority to monitor the increasing impact of MP contamination.^8^

## Microplastics in Seafood: Exposure and Potential Effect on Human Health

MP exposure through dermal contact is considered a less significant source, mostly related to plastic monomer and additive exposure, among the different human exposure routes, while individual inhalation amounted to 26–130 airborne MPs/day.^24^ The main pathway of human exposure is the ingestion of food with an estimated intake of 39,000–52,000 plastic particles/person/year, of which 37–1000 are from sea salt, 4000 are from tap water, and 11,000 particles/person/year are from shellfish.^24^ Moreover, as an additional exposure pathway, the atmospheric fallout of plastic fibers during food production should be investigated.^24−26^

The human health effects of ingested MPs may be caused both by plastic particles and by their additives or adhering contaminants.^12,27^ It is not clear if MPs remain in the gut lumen after ingestion or can translocate across the gut epithelium. Gut cells may absorb particles of a few microns, while MPs up to 10 μm may be detected by Peyer’s patch cells of the ileum. MPs of a size of 130 μm can translocate in tissue through paracellular transport in the form of persorption and determine a systemic exposure.^26^ The translocation to secondary target organs and tissues (e.g., lymphatic system) has been demonstrated in humans (particle sizes of 160 nm to 150 μm), rabbits (100 nm to 10 μm), dogs (3–100 μm), and rodents (10 nm to 40 μm).^2,25,28^ Plastic particles (>0.2 μm) are removed from the lymph into the gut through the splenic filtration, while those in the blood are eliminated by bile and excreted via faeces.^25,28^

MP human exposure may induce physical and chemical toxic effects. The type of particles and individual susceptibility may influence the adverse effects. The physical effects may have different concerning impacts, including enhanced inflammatory response, oxidative stress, cell damage, and size-related toxicity.^3,26^ Furthermore, the immune system is not able to eliminate the plastic particles, and consequently, chronic inflammation and an increase in the risk of neoplasia may occur.^24^ Regarding the chemical effects, it is known that MPs may transfer different chemicals, such as compounds intentionally added during the manufacturing process, and environmental contaminants as toxic metals, polychlorinated biphenyls (PCBs), and PAH.^5^ Among the plastic additives, bisphenol A (BPA) and phthalates are endocrine disruptors and can induce carcinogenic and neurotoxic effects on animals and humans.^5,25,29^ The exposure to different chemicals may occur directly through MPs or also by the consumption of fish which previously ingested MPs accumulating in their tissue the chemicals.^8^ The average consumption of 225 g of mussels without shells containing 4 particles/g (the highest number of MPs detected) might induce the ingestion of about l.0 g of plastics. In this scenario, the ingestion of MPs has little influence on the exposure to PCBs, PAHs, and BPA.^2^ However, given the uncertainties of data on the occurrence of MPs in food, the risk for human health related to seafood consumption is still unclear.^26^ Most of the information on MPs in the marine food web concern their occurrence in the GI of seafood, even if this part is normally discarded before the consumption.^2−5^

Moreover, among the potential effects on human health, microbiological risk linked to the MP ingestion should be considered, since microorganisms and invertebrates may colonize plastic particles. MPs might favor the long-range transport of alien species or, also, act as reservoirs for pathogen transmission.^3−5,30^

## Perspectives on Microplastics Research and the Implication for Food Safety and Health

The increasing production of plastic associated with inadequate management of plastic waste is the main factor influencing the MP diffusion in the environment. The ubiquity of MP pollution, including the Arctic, Antarctica, the deep ocean, and secluded mountainous regions, has increased the concern about negative physiological (e.g., growth, reproduction, mortality) and behavioral (e.g., feeding) impacts on marine biota as well as their occurrence in foodstuff.^27^ MP monitoring is one of the objectives of the European Marine Strategy Framework Directive. The achievement of Good Environmental Status for the marine environment has been recommended by 2020 to the EU Member States. They shall establish a list of species to assess the extent of litter and microlitter contamination through regional or subregional cooperation (Commission Decision 2017/848/EU).^15^

The EFSA reports have highlighted that the MP bioavailability through the human food web represents a potential risk for human health.^2^ However, data on the occurrence of MPs in the environment and seafood are uncertain and incomplete for an appropriate risk assessment. Data gaps such as the use of standard sampling protocols, collection of a significant number of representative samples of a specific marine area/population, choice of a suitable species as an indicator to monitor MP pollution, and human health effects in seafood have been identified.^1,2,8,11^ Hence, one of the future challenges will be the development of standardized monitoring methods and protocols to harmonize laboratory procedures for MP analysis.^7,8^ Their use will allow the comparison of disposable data on the occurrence of plastics in marine biota and the risk assessment for human health.^8^

Traceability of the fate of MPs in contaminated seafood is essential to understand their bioaccumulation and biomagnification in the marine environment. Without appropriate knowledge of the MP diffusion degree from the preys to the predators, the evaluation of the effects of eating seafood is difficult.^27,28^

A comprehensive assessment should consider not only the MP levels but also the concentrations of MP contaminants along the food chain and the impact that cooking or other food processes may have on their desorption and subsequent bioaccessibility.^28^ In this view, also the scientific debate should be focused on both the concern about the MP environmental pollution and the toxic effects of additives and plasticizers used during plastic production.^25^

Considering the increasing occurrence of MPs in the environment, plastic pollution is of concern because may also influence food security, food safety, and human health. From a future perspective, the risk assessment framework should be based on a harmonized protocol including techniques and methods for MP analysis in environmental matrixes and living organisms. Hazard and risk assessment should be carried out involving terrestrial and freshwater ecosystems also. Moreover, given the limited data, further studies are needed to evaluate how the exposure to MPs poses a risk for human health.
